# canSAR: update to the cancer translational research and drug discovery knowledgebase

**DOI:** 10.1093/nar/gky1129

**Published:** 2018-11-28

**Authors:** Elizabeth A Coker, Costas Mitsopoulos, Joesph E Tym, Angeliki Komianou, Christos Kannas, Patrizio Di Micco, Eloy Villasclaras Fernandez, Bugra Ozer, Albert A Antolin, Paul Workman, Bissan Al-Lazikani

**Affiliations:** 1 Department of Data Science, The Institute of Cancer Research, London SM2 5NG, UK; 2 Cancer Research UK Cancer Therapeutics Unit, The Institute of Cancer Research, London SM2 5NG, UK

## Abstract

canSAR (http://cansar.icr.ac.uk) is a public, freely available, integrative translational research and drug discovery knowlegebase. canSAR informs researchers to help solve key bottlenecks in cancer translation and drug discovery. It integrates genomic, protein, pharmacological, drug and chemical data with structural biology, protein networks and unique, comprehensive and orthogonal ‘druggability’ assessments. canSAR is widely used internationally by academia and industry. Here we describe major enhancements to canSAR including new and expanded data. We also describe the first components of canSAR*black*—an advanced, responsive, multi-device compatible redesign of canSAR with a question-led interface.

## INTRODUCTION

Translating biological discoveries into the clinic requires the analysis and support of vast amounts multidisciplinary data that is generally difficult to integrate and maintain. canSAR (http://cansar.icr.ac.uk) (1–3) is a freely available, multidisciplinary knowledgebase developed to bring together billions of experimental datapoints and information in order to empower translational research and drug discovery. canSAR not only collates, but also integrates inter-disciplinary data including genomic, transcriptomic, protein, pathway, chemical, pharmacological and 3D structural data. Moreover, canSAR is also the world's most comprehensive druggability assessment resource. canSAR provides a powerful, unique and user-friendly portal to help generate and test hypotheses and, in particular to support scientific decision-making in drug discovery both before and after target selection. While some of the data in canSAR, in particular genomic and functional biological screen data, are specific to cancer, much of the data within it are relevant to other diseases.

To our knowledge, canSAR is the first and remains the largest, most comprehensive, multidisciplinary resource to support translational cancer research and drug discovery. Since its first release in 2011, canSAR has been used by over 200,000 unique visitors from 200 countries (source: Google Analytics, excluding IP addresses from our home institute). To date, canSAR and its methodologies has been cited by well over three hundred peer-reviewed publications.

Here, we describe the most up to date version of canSAR, canSAR v4.0. In addition, we describe the first components of canSAR*_black_* —the ‘next generation’ of canSAR, comprising an advanced, responsive, multi-device compatible redesign of canSAR with a question-led user interface. canSAR*_black_* is named after the pharmacologist Sir James Black, an early exponent of developing drugs against a specific molecular target. Table 1 summarizes key differences between the previous v3 version and the current version of canSAR.

**Table 1.** Comparison of data and features between v3 and current version

Feature	canSAR v3	canSAR v4/Black
**DATA**		
Protein structures	>111 000 protein structures for c297 000 individual PDB chains	>140 000 protein structures and >390,000 individual PDB chains
Data on small molecules	Over one million, bioactive, small molecule drugs and compounds corresponding to >8 million pharmacological bioactivities	Integration of ChEMBL 24 and canSAR-curated small molecules, totaling over 1.9 million drugs and chemical compounds and >15 million pharmacological bioactivities
Curated protein-protein interaction network	Network of c13 000 proteins	Network of c14 000 proteins
Information on clinical trials	Integration of over 179 000 clinical trial summaries	Integration of over 228 000 trial summaries
Integration of expert-curated assessment of chemical probes	-	Full integration of ProbeMiner
Druggability assessments of protein complexes and ligandable interface cavities	-	Annotation of >207 000 biological complexes and identification of 77 000 ligandable interface cavities
Normal tissue gene expression reference	-	Approximately 10 000 samples from GTEx
Post-translational modification data	-	Curated data from Phosphosite
Cancer association information	-	Novel analysis of each gene's association with different cancer types based on clinical studies, patient mutation, copy number alteration, gene expression and cell line dependency data.
Deeper annotation of patient omic data	-	Pathology- and clinical based staging curated, omic profiles organized into the different stages
**FUNCTIONALITY**		
Search features	Basic search facility	New responsive Elastic Search-based search engine
Druggability view	V3 Table with summary of structure-based druggability for individual chains	Completely re-implemented, multilayered druggability and ligandability. Users can explore all alternative druggability assessments. For structure-based druggability, expert users can drill deeply into the analysis to individual cavities on individual structures.
Cancer association view	-	Visualization of key cancer-type association. Users can explore details of associations based on clinical studies, patient omics and cell line dependencies

## DATA CONTENT AND GROWTH

canSAR v4.0 contains updates to many key datasets, together with integration of new resources. Gene expression and mutational data have been updated using The Cancer Genome Atlas (TCGA, GDC Version 11), including 2.8 million protein coding mutational events in cancer patients of which 425,000 are from cancer metastases (4). Where matched normal tissue profiling is available, these data are also curated for comparison. Gene expression data are similarly curated from TCGA. In order to provide researchers with normal tissue reference points, canSAR now additionally integrates profiling data on ca. 10,000 samples from the GTEx project (https://gtexportal.org/ (5)).

canSAR fully incorporates and integrates a number of medicinal chemistry databases, as well as an increasing number of canSAR-curated small molecule compounds. Collectively, these provide canSAR with over 1.9 million drugs and chemical compounds and >15 million pharmacological bioactivity data points. canSAR continues to update its content weekly, including curation and analysis of 3D protein structure data. It currently holds >140 000 protein structures (>390 000 individual PDB chains).

To enable more detailed investigation of proteins, canSAR is compiling and curating posttranslational modification data from public resource and the literature, including residue-level phosphorylation data from Phosphosite (6). canSAR now fully integrates these data to enable enhancements of directional protein-protein interaction data, such as kinases and their substrates. Furthermore, this information can help researchers select potential target engagement and mechanistic biomarkers for kinase activity in the laboratory.

We increased the curated canSAR protein-protein interactome to cover almost 14 000 proteins. Additionally, canSAR now contains details of over 228,000 clinical trials from ClinicalTrials.gov, which are linked to disease, protein targets and compounds.

## ENHANCEMENTS AND NEW DATA IN DRUGGABILITY ASSESSMENTS AND ANNOTATION

canSAR provides a comprehensive suite of sophisticated, orthogonal druggability assessment methodologies utilizing machine learning and AI predictions developed by the team (2,7,8). We identify and predict the ‘ligandability’—the propensity of a protein to bind a drug-like small molecule—of proteins with known 3D structure (2). As mentioned above, the canSAR 3D pipeline is updated weekly and to date has identified and analysed >3 777 000 cavities on >140 000 protein structures (>390 000 PDB chains). New to canSAR v4.0, we now identify and annotate biologically relevant structural complexes in the PDB (9), and identify potential ligandable cavities at their interfaces. We have analysed >207,000 biological complexes and identified 77 000 ligandable interface cavities.

Additionally, we include prevalent somatic mutations and sequence conservation across families to annotate potentially druggable non-primary sites, that may lead to the identification of important, pharmacologically accessible allosteric or second sites.

To allow orthogonal assessment of potential targets, and also enable the assessment of targets that do not have a 3D structure available, we continue to enhance and update the non-3D protein structure-dependent druggability assessments. We have updated the chemistry-based assessment (7) which evaluates targets based on the chemical landscape of the target itself and its protein family. Compounds active against either the target in question or its homologues are assessed for their potency, ligand-efficiency, diversity and drug-likeness, among other parameters. In canSAR v4.0, we provide chemistry-based assessment for >8300 human proteins. canSAR also includes an AI-based assessment of ‘drug target-likeness’ that is based purely on the protein's behaviours in cellular networks (8). This assessment examines whether a protein exhibits behaviours (in terms of its short and long-range protein-protein interaction patterns) characteristic of target of cancer drugs and/or a target of drugs from non-cancer therapeutic areas. canSAR now contains these assessments for almost 14 000 human proteins.

**Figure 1.**
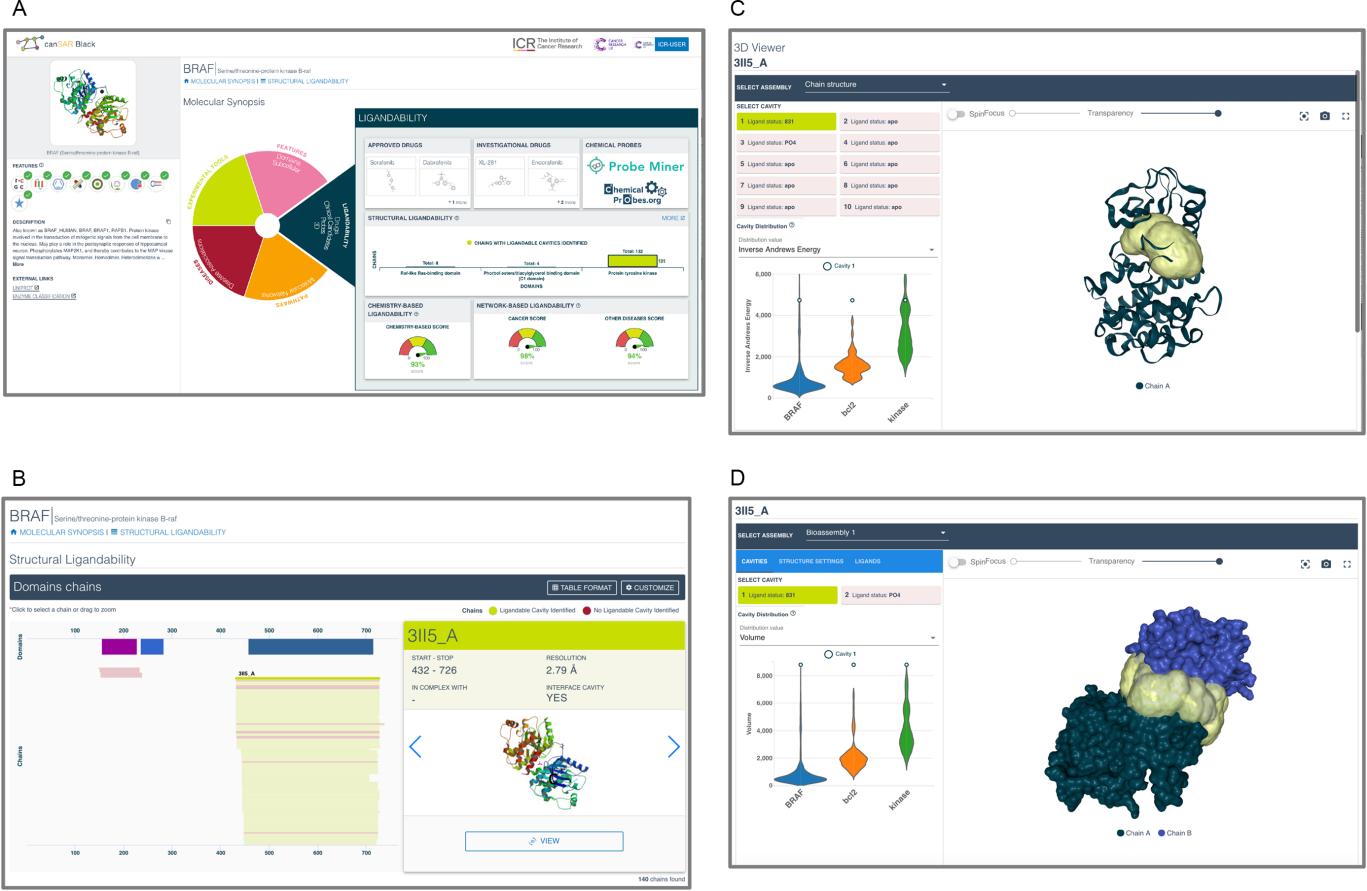
canSAR*_black_* target synopsis showing the navigation wheel and druggability assessment. (**A**) Target synopsis. Left hand panel displays the icons representing key information on the target as well as a summary description of it. The icons are ticked green and coloured if the target meets certain criteria. For example, if the target is a drug target; if it has a structurally ligandable cavity; if it has bioactive compounds etc. Hover-over of the icons provides the user with the information on each. The right-hand panel provides the new navigation wheel. The user can navigate to key sections of information by clicking on the appropriate sector in the wheel. Here the ligandability information is summarised. First, displaying drugs that are approved or under investigation as well as links to chemical probes; second, summarizing the structurally characterised domains of the protein with a histogram showing the number of available structures for that domain; and whether they contain ligandable cavities; third, the bottom layer provides the chemistry-based assessment and network-based target likeness as a ‘meter’ showing how high the score is for the particular target. (**B**) Users can navigate into more detailed information about the chains and domains and navigate through all individual structures. (**C**) Detailed view of individual structures and cavities. Users can explore each of the key properties of these cavities and compare them to known drug target cavities such as kinases and BCL2. (**D**) Ligandable cavities at protein interfaces can be examined in the same way. All images and raw data are downloadable.

Together, these unique data make canSAR the largest public resource for druggability assessment and provide a powerful enabler for target selection and validation for drug discovery. Examples of how these data can be viewed are shown in Figure 1.

## ENHANCED CURATION OF PUBLIC DATA FOR MORE MEANINGFUL INTERPRETATION

Integrating interdisciplinary data into canSAR has already proved to be very useful for canSAR users who can examine clinical genomics, pharmacological data and biophysical data on one target protein at a time. However, in order to further empower researchers in generating biological hypotheses and devising their next experiments, continuing deeper curation of these integrated data is essential. In addition to the curation described in previous canSAR publications (1–3), we now deeply curate and re-analyse all gene expression data from TCGA and GTEx to enable cross-platform and cross-study comparisons.

**Figure 2.**
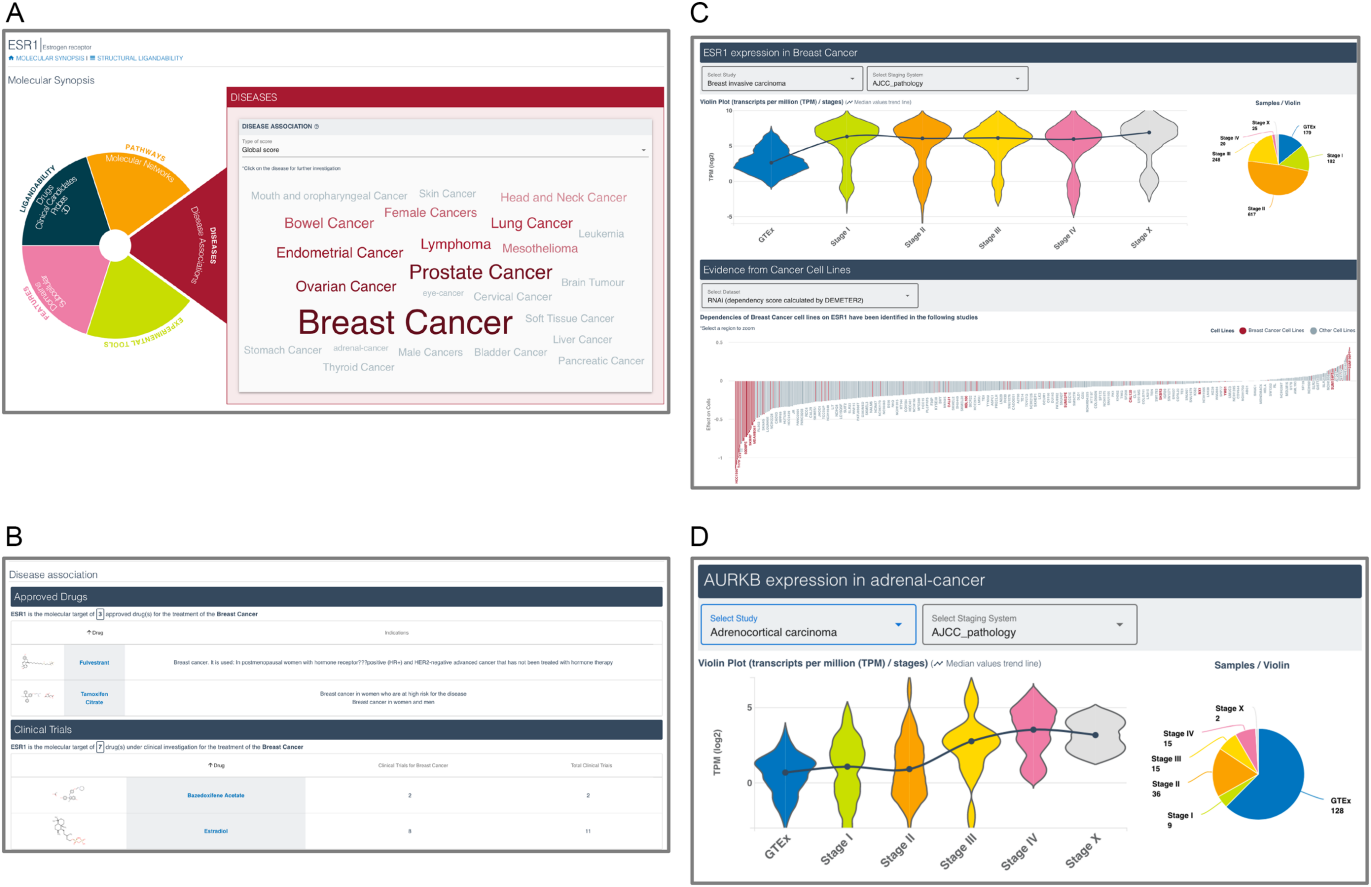
canSAR*_black_* target synopsis showing the cancer association summary. (**A**) word-map showing the association of each broad cancer type with the target. The size of the name corresponds to the association score of the target with this cancer. (**B**) Where drug information is available, drug approval and clinical trial information are listed. (**C**) Gene expression data and cell-line dependency are among the detailed information provided to help the user examine the evidence of disease association. (**D**) Gene expression changes of AURKA with progression of clinical stage in adrenal carcinoma.

In order to provide more meaningful annotation, we have curated and standardised staging systems for the majority of samples in canSAR. We primarily adopted the TCGA pathology-based system where available and appropriate. Both clinical and surgical-based information on the cancer at the date the biopsy was taken were combined. This allows tracing the aberrations of genes not just between studies and normal tissue, but also between different stages of progression of the disease (Figure 2). In some cases, the use of the clinical staging information was less relevant, in which case we adopted the most appropriate staging system (e.g. Gleeson for prostate cancer). In each study, we map the molecular profiling data to these stages and provide the user with the precise staging system used.

Another important enabler for researchers is the use of chemical probes. Probe Miner™ (probeminer.icr.ac.uk (10)) is a large-scale, objective chemical probe assessment platform which applies assessment of probe fitness factors to all compounds in canSAR, and ranks these compounds for their suitability to be used as chemical probes for any specific human protein. Probe Miner was developed to be a live-data-responsive and automated probe assessment tool, to complement the expert annotation of chemical probes provided by the Chemical Probes Portal (chemicalprobes.org). Between the two resources, researchers can select the most appropriate probes for their research. canSAR now contains the probe annotations from both resources and provides links to them, where available, for each human protein.

## canSAR_*BLACK*_ NEW INTUITIVE INTERFACE FOR TRANSLATIONAL RESEARCH

canSAR is evolving and increasingly focusing on user experience and key capabilities. canSAR_*black*_ is a new interface to canSAR, being designed with the improving user experience as a central driver. It provides a more advanced interface with responsive, interactive visualizations, filtering and navigation capabilities. canSAR*_black_* works across all platforms and is mobile device-compatible and screen-resolution adaptive. The main interface development tool is a javascript framework, Vue.js (https://vuejs.org/) using Vuex pattern (https://vuex.vuejs.org/).

Over the next year, all canSAR functionality will migrate to the canSAR*_black_* interface. Meanwhile, in response to user feedback, we have focused on the development a better search facility and a new version of the Target Synopsis. A key paradigm change in canSAR*_black_* is the provision of data as answers to key translational questions such as: what is the evidence that a target might be suitable for drug discovery?; what is the evidence of a target's association with a particular cancer?; what are the key experimental tools available to mechanistically explore a target? Moreover, canSAR*_black_* provides multi-layered summaries. The first layer is a simple visual summary of the key information. Users can then navigate into increasingly complex visualizations and data analyses in accordance with their interest and expertise levels in a specific area. Two examples of these are illustrated below and in Figures 1 and 2.

## MULTI-LAYERED TARGET DRUGGABILITY ASSESSMENT VIEW

Druggability assessment allows users to examine a target's potential for drug discovery, identify key risks associated with the pursuit of the target, and devise experiments to address these risks. canSAR*_black_* provides this information in a multi-layered approach to allow expert users to navigate and examine the data in greater depth than was previously possible, while maintaining an understandable summary view for users with less experience in these areas.

Initially, a summary of all the information is provided on the target (Figure 1A). This includes known drugs acting on the target where available, together with a summary of the level of structural characterisation of the protein with the PDB structures coloured according to the existence of druggable domains. The chemistry- and network-based assessments described above are also provided. To improve understanding, the target is benchmarked against other human proteins in each assessment. A meter-view provides a high-level instant indication of the potential of this target. To explore the information in detail, users can access the next layer of structural annotation (Figure 1B). Here, the user can navigate through images and ligandability scores for each individual structure.

Moreover, canSAR’s druggability assessment utilizes a large number of features in its predictive algorithm, including volume, enclosure and additional features such as hydrogen-bond donors, hydrophobic fraction etc. These features are useful for expert users when examining novel potential targets. We provide distributions, in the form of violin plots, of how the target in question performs when compared to bona-fide drug targets. Specifically, we provide comparator distributions for the druggable cavities of highly druggable targets (kinases) and also more challenging protein–protein-interaction druggable targets represented by BCL2. These comparators allow users to gain a rapid understanding of how their target compares with typical drug targets.

Expert users can navigate to even deeper layers of information (Figure 1C). Here, users can examine each individual cavity in detail, look for key residues (e.g. cysteines for covalent interactions) and download all detailed data. Where cavities are also identified in biological complexes, users can explore these with the same level of detail through the drop-down menu and selecting one of the bioassemblies (Figure 1D).

## MULTI-LAYERED CANCER-ASSOCIATION VIEW

We developed a scoring system that associates a gene with a particular cancer. The score takes into account all available information from the clinic – such as drug approvals and clinical trials where available; any aberrations or alteration of the gene in patient data; and dependency on the gene in cancer cell lines. The total score is used to summarize the relevant cancers using a word map paradigm (Figure 2A). Clicking on any one cancer type in the word map leads the user to the details page that provides the key information in each of these areas (Figure 2B and C).

A key advantage of the staging curation referred to above is that users can now compare cancer gene expression data against normal tissue from GTEx as well as progression among pathological or clinical stages of the. For example, the expression of the kinase AURKA increases with the more advanced stages of the cancer as shown in Figure 2D.

## CONCLUDING REMARKS AND FUTURE DEVELOPMENT

Currently, key canSAR*_black_* functionality is accessed through links in canSAR v4.0. canSAR will transfer to the new canSAR*_black_* interface over the next calendar year with regular updates to the site. We will enhance the data in canSAR including unique-to-canSAR data through abstraction of key literature on chemical probes; biological activities; target engagement biomarkers; and drug combinations. Moreover, canSAR will contain new chemical analysis pipelines to enable more sophisticated analysis and interrogation of its chemistry data. Close links are provided to a range of other key resources, including The Chemical Probes Portal and Probe Miner for chemical probes (10) and the Cancer Dependency Map (DepMap) resource that connects tumour features with tumour dependencies (11) (https://depmap.org). This will enable users to seamlessly navigate between canSAR and DepMap for their targets of interest. Moreover, canSAR drug and druggability assessment algorithms will expand substantially to cover biologics, immunotherapeutics and cryptic druggable sites.
